# Case report of homozygous E200D mutation of *PRNP* in apparently sporadic Creutzfeldt-Jakob disease

**DOI:** 10.1186/s12883-021-02274-w

**Published:** 2021-06-28

**Authors:** Ahamad Hassan, Tracy Campbell, Lee Darwent, Hans Odd, Alison Green, John Collinge, Simon Mead

**Affiliations:** 1 Department of Neurology, Leeds General Infirmary, Leeds, LS1 3EX UK; 2 MRC Prion Unit at UCL, UCL Institute of Prion Diseases, 33 Cleveland Street, London, W1W 7FF UK; 3 National Prion Clinic, University College London Hospitals NHS Foundation Trust, London, UK; 4 National CJD Research & Surveillance Unit, Centre for Clinical Brain Sciences, University of Edinburgh, Edinburgh, EH16 4SB UK

**Keywords:** CJD, Prion, PRNP, Inherited prion disease, Recessive, E200D

## Abstract

**Background:**

Inherited prion diseases are rare autosomal dominant disorders associated with diverse clinical presentations. All are associated with mutation of the gene that encodes prion protein (*PRNP*). Homozygous mutations with atypical clinical phenotypes have been described but are extremely rare.

**Case presentation:**

A Chinese patient presented with a rapidly progressive cognitive and motor disorder in the clinical spectrum of sCJD. Investigations strongly suggested a diagnosis of CJD. He was found to carry a homozygous mutation at *PRNP* codon 200 (E200D), but there was no known family history of the disorder. The estimated allele frequency of E200D in East Asian populations is incompatible with it being a highly penetrant mutation in the heterozygous state.

**Conclusion:**

In our view the homozygous *PRNP* E200D genotype is likely to be causal of CJD in this patient. Homotypic PrP interactions are well known to favour the development of prion disease. The case is compatible with recessively inherited prion disease.

## Background

Prion diseases are rare and transmissible neurodegenerative disorders associated with accumulation of assemblies of misfolded prion protein (PrP). Over 85% of the annual incidence of human prion disease takes the form of sCJD, typically presenting as a rapidly progressive dementia with ataxia, myoclonus and motor problems. Inherited prion diseases (IPD) comprise the large majority of the remainder of cases, which have a wide variety of clinical presentations, sometimes indistinguishable from sCJD, but often with much slower clinical change [1]. All IPD is associated with coding mutation of the gene that encodes prion protein (*PRNP*) and a large number of variants have been described. IPD segregates in families as an autosomal dominant trait, although some variants have relatively late clinical onset and carriers may die from other conditions before the disease manifests. The most common mutation that causes IPD, E200K, has a clinical presentation that is hard to distinguish from sCJD. E200K is frequent in particular regions of the world, and a small number of patients with a homozygous E200K mutation have been seen, having a slightly earlier onset of disease compared with heterozygous patients, and some altered clinical features [2]. A patient with a homozygous Q212P mutation has been reported. Here we present and discuss the case of a patient with CJD and a homozygous E200D mutation, not previously described.

### Case presentation

A 61 year old man of Chinese ethnicity with no significant past medical history presented with a 6 week history of behavioural change including aggression, poor memory, praxis problems, slurred speech and loss of balance. There was a recent history of weight loss and vomiting. On examination he appeared to understand and speak English but the content of his speech was disorganised and he appeared confused. Examination revealed normal eye movements. He was dysarthric and demonstrated ataxia in all four limbs with truncal ataxia also. There was no myoclonus, or pyramidal or extrapyramidal signs at this stage.

The patient’s father died aged 61 of a non-neurological condition, the status of the patient’s mother is not known. There were no prior diagnoses of CJD or dementia in the wider family known to the patient’s children.

**Fig. 1:**
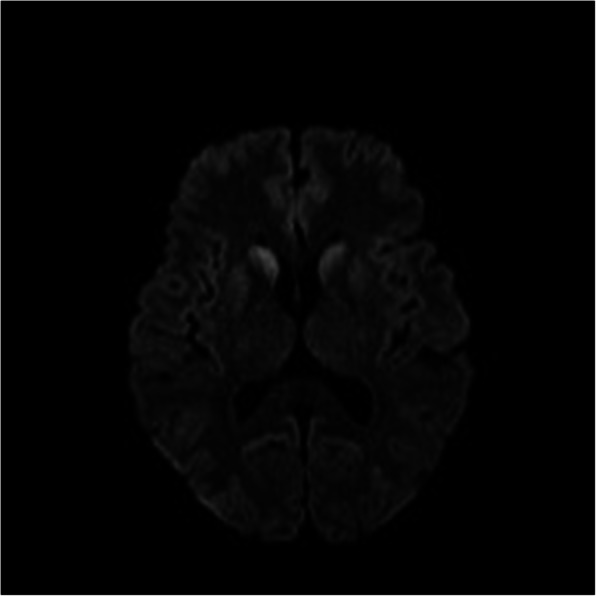
Axial diffusion weighted image showing bilateral high signal in the striatum and cortex

**Fig. 2:**
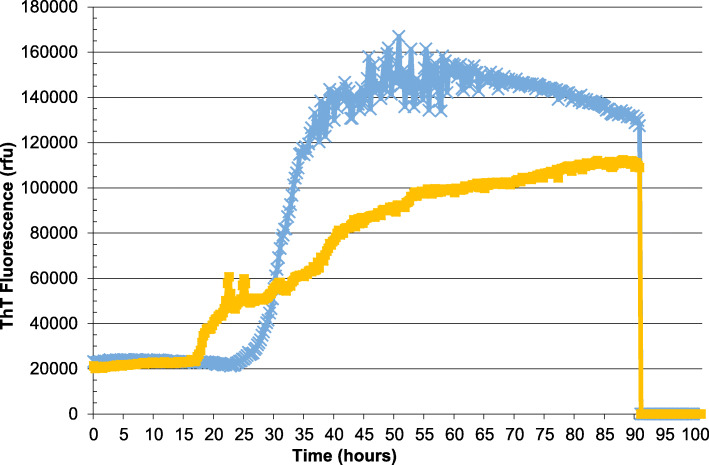
CSF RT-QuIC trace of reaction seeded with 30 μl of CSF (blue) and 15 μl (orange). Results expressed as the mean of the two highest of four replicates

Initial work-up showed normal full blood count, standard biochemical profile, B12 and folate. Paraneoplastic, VGKC and anti-NMDA antibodies were negative as were HIV, syphilis and hepatitis serology. EEG was reported as mild slowing only, with no periodic sharp wave complexes. A brain MRI showed abnormal symmetric restricted diffusion in the corpus striatum bilaterally and cortical diffusion restriction in the frontal lobes and around the parieto-occipital fissure bilaterally (Fig. 1). CSF was acellular, with normal protein and glucose. CSF Viral PCR was negative but 14–3-3 and S-100b were abnormal. A diagnosis of CJD was confirmed by a positive reaction when CSF was analysed in quadruplicate by RT-QuIC (real-time quaking induced conversion, Fig. 2). The E200D mutation was detected by bidirectional sequencing and was linked to a methionine homozygous genotype at polymorphic codon 129.

Over the course of his admission his neurological state rapidly worsened. He began to exhibit startle responses and multifocal myoclonus. Later he became increasingly agitated with distressing visual hallucinations which were managed with lorazepam. In the final days of his illness he became mute and bed bound with generalised spasticity. Following discussion with his family palliative care was commenced. Death was certified within 4 weeks of admission (total duration 10 weeks).

## Discussion and conclusions

Although no post-mortem examination was done the diagnosis of this patient is almost certain. The MRI findings and RT-QuIC test are both highly specific in isolation. We are not aware of a single report in the literature of a false positive diagnosis when both of these tests are abnormal. The E200D variant has been found in the East Asian population with an allele frequency of approximately 1 in 10,000 (based on 2 occurrences in 18,394 East Asian exomes reported on gnomAD) [3]. As the variant has not been reported in the heterozygous state in any CJD patient, it is highly likely to be benign as this genotype.

The homozygous E200D genotype has never been observed in population databases. It should be extremely rare and found predominantly in families with some degree of consanguinity, which was possible but not certain in this case. Family history is a good guide to help decision making about the penetrance of novel *PRNP* variants, however in this case we would not expect a family history as the parents are expected to be heterozygous carriers of a benign variant. In our opinion, it seems likely that the homozygous E200D genotype was causal in this patient, the alternative possibility is of a coincidental occurrence of sCJD with a genotype of *PRNP* never before observed [4]. The situation seems similar to the Q212P variant of *PRNP* which appears to have low penetrance in the heterozygous state, but has caused an atypical early onset prion disease in the homozygous state [5].

## Data Availability

All data is available on reasonable request to the corresponding author.

## Abbreviations

CJD: Creutzfeldt-Jakob disease
PRNP: Prion protein gene
VGKC: Voltage-gated potassium channel
NMDA: N-Methyl-D-aspartic acid
RT-QuIC: Real-time Quaking Induced Conversion Assay
PCR: Polymerase chain reaction
